# Corrigendum to “NUDT21‐mediated Alternative Polyadenylation of CDK19 Reprograms Cholesterol Biosynthesis to Drive Colorectal Cancer Progression”

**DOI:** 10.1002/advs.77060

**Published:** 2026-08-13

Y. Yu, J. Ma, M. Zhou, et al. “NUDT21‐mediated Alternative Polyadenylation of CDK19 Reprograms Cholesterol Biosynthesis to Drive Colorectal Cancer Progression,” *Advanced Science*, 13, no. 7 (2026): e18346, https://doi.org/10.1002/advs.202518346.

In paragraph 2 of the “Result 2.5. NUDT21 Lengthens the 3′UTR of CDK19 to Regulate Cholesterol Biosynthesis and CRC Proliferation” section, an inadvertent figure assembly error was identified in Figure S7A. The image representing the cell viability curve of HCT116 cells treated with indicated concentrations of SEL120‐34A for 72 h (*n* = 5) was inadvertently replaced. Please find the corrected figure below.


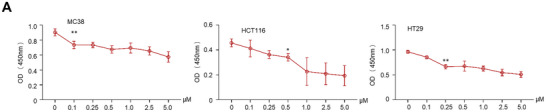


**FIGURE S7** | CDK19 promotes CRC cell growth in vitro and directly acts on key cholesterol synthesis genes. (A) Cell vitality curve of HCT116, HT29, and MC38 cells treated with indicated concentrations of SEL120‐34A, a CDK19 inhibitor, for 72 h (*n* = 5). Data are presented as mean ± SEM. *p < 0.05, **p < 0.01 by one‐way ANOVA (A).

In addition, the statistical significance annotation (asterisk, *) in the bar graph of Figure 5H was inadvertently omitted during figure preparation. The figure has been corrected to include the appropriate statistical significance annotation.


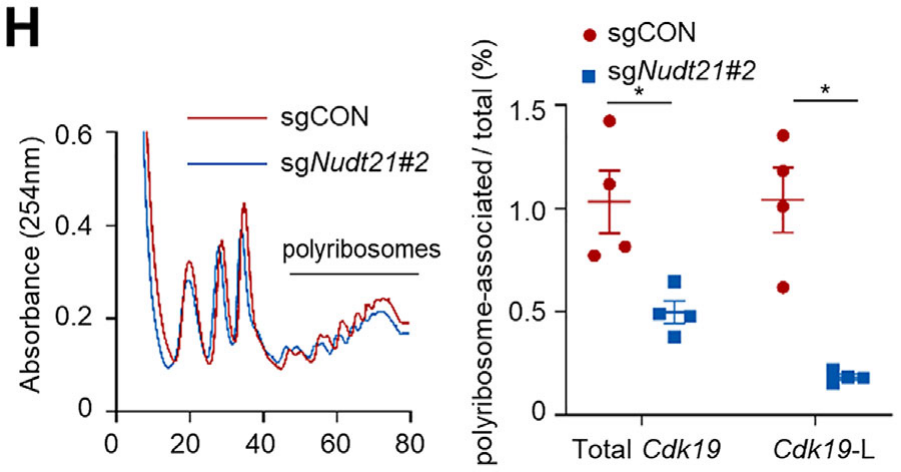


We apologize for this error.

